# Supplementary figures and images for: Aedes albopictus bionomics data collection by citizen participation on Procida Island, a promising Mediterranean site for the assessment of innovative and community-based integrated pest management methods
Source: PLoS Negl Trop Dis. 2021 Sep 16;15(9):e0009698. doi: 10.1371/journal.pntd.0009698 (PMC8445450; doi:10.1371/journal.pntd.0009698)

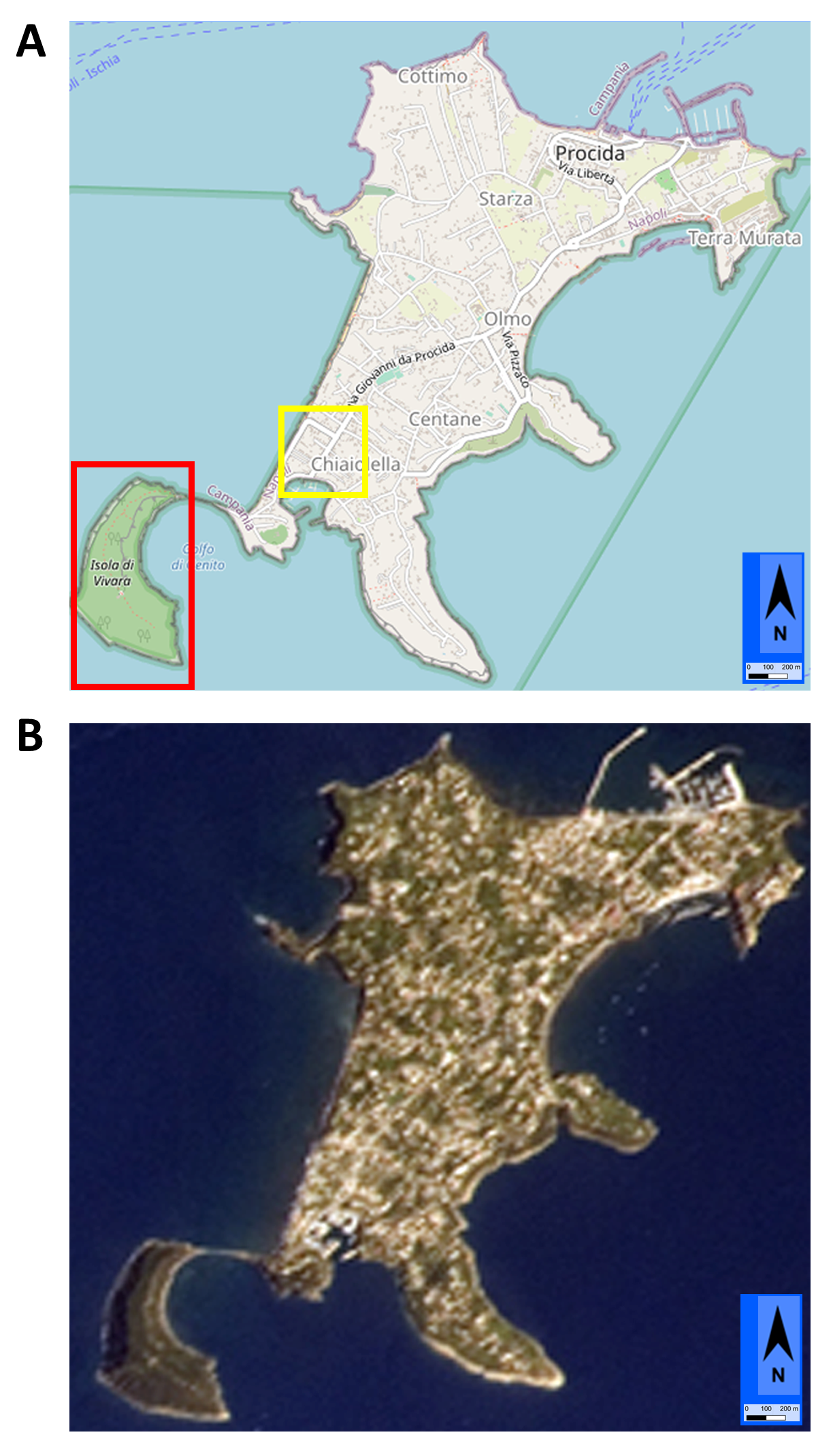

Supplement: S1 Fig — A) Procida Island map. Yellow box shows the extent of map showed in Fig 5, red box shows the extent of map showed in S5 Fig. The base layer of the geographic background map has been sourced from an open maps access (https://glovis.usgs.gov/app). B) Procida island satellite picture (https://eol.jsc.nasa.gov/SearchPhotos/). In both representations of the island, a clear uniformity in the territory organization is visible. Most of the Procida territory is organized in private properties with residential buildings surrounded by green areas, including gardens with ornamental flowers, vegetable cultivations and/or orchards with citrus plants and family-type farming of chickens and rabbits. The only exception is represented by the Vivara natural reserve, which is inhabited by humans and connected with the main island by a concrete bridge (150m long). (TIF) [file pntd.0009698.s001.tif]

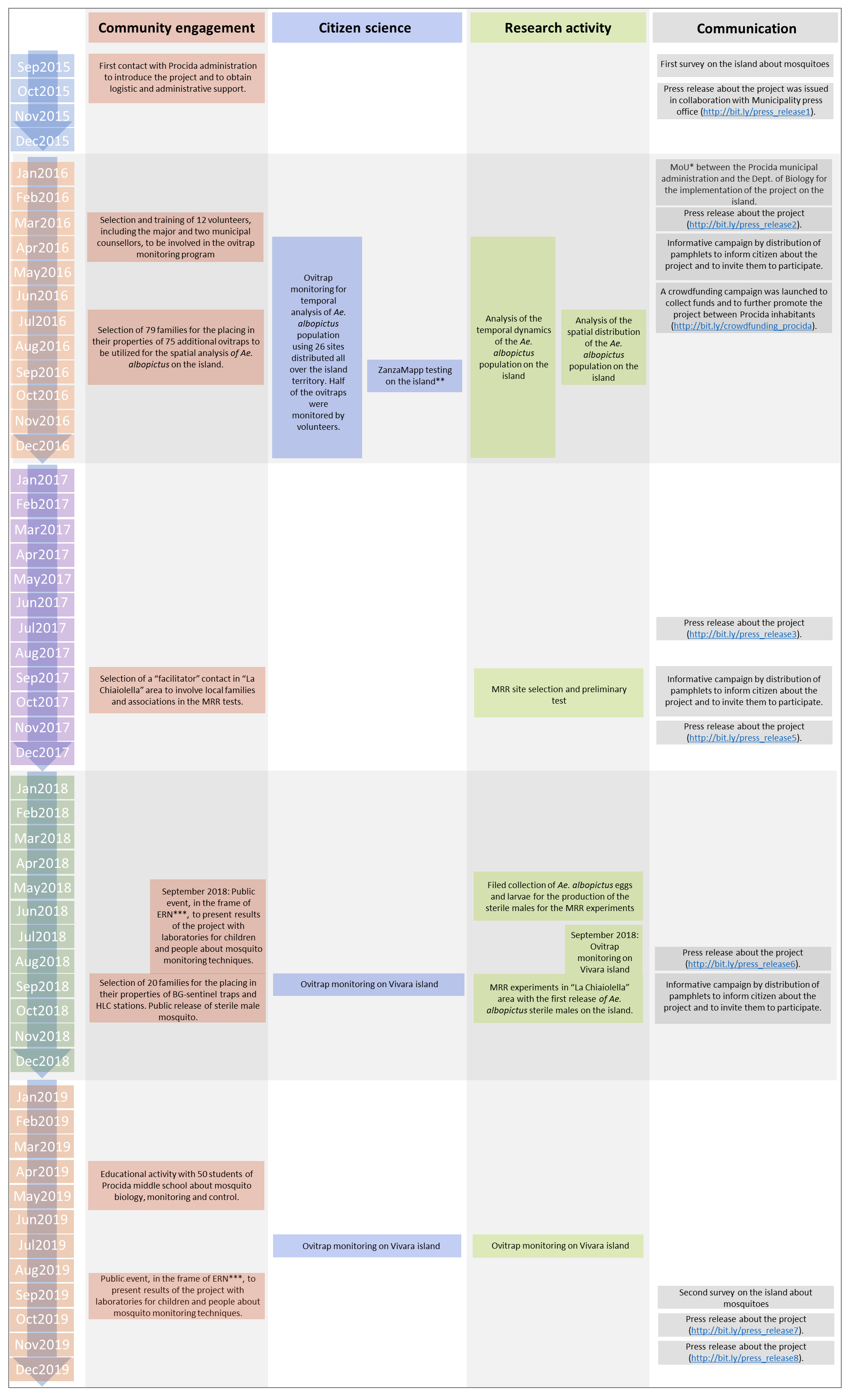

Supplement: S2 Fig — (*) MoU = Memorandum of Understanding. (**) ZanzaMapp is a mobile app for mosquito monitoring (https://www.zanzamapp.it/) [93] that was tested on Procida island during September 2016. (***) ERN = European Research Night. The public activities were organized on Procida island in the frame of the MEETmeTONIGHT project (http://www.meetmetonight.it/) funded by EU. (TIF) [file pntd.0009698.s002.tif]

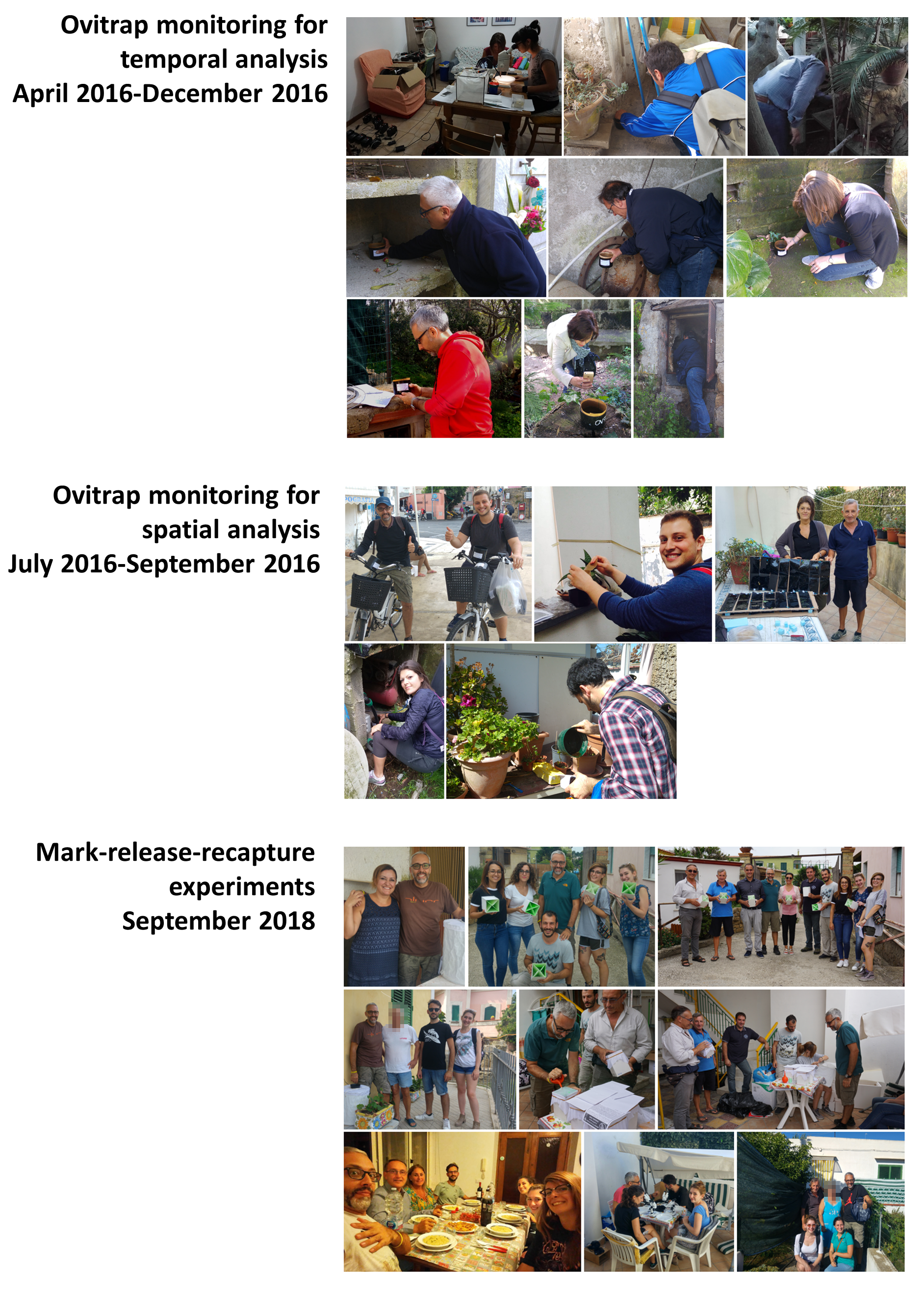

Supplement: S3 Fig — All the people (co-authors of the present manuscript and Procida volunteers) present in this figure gave their written consent to be photographed and to have their images published under a creative commons license. (TIF) [file pntd.0009698.s003.tif]

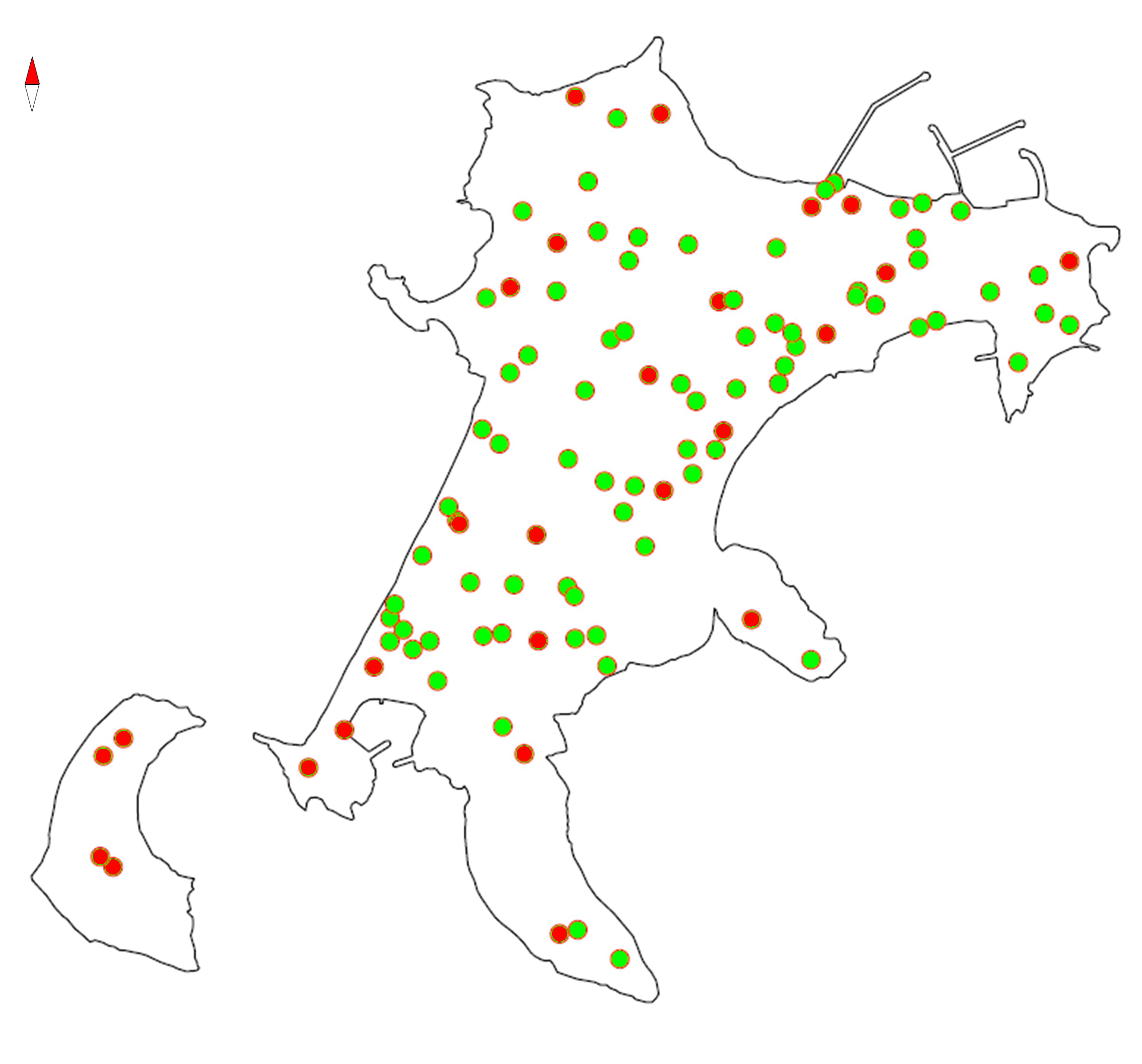

Supplement: S4 Fig — The position of the 101 ovitraps utilized for temporal (red) and spatial (red and green) analyses are reported. The base layer of the geographic background map has been sourced from an open maps access (https://glovis.usgs.gov/app). (TIF) [file pntd.0009698.s004.tif]

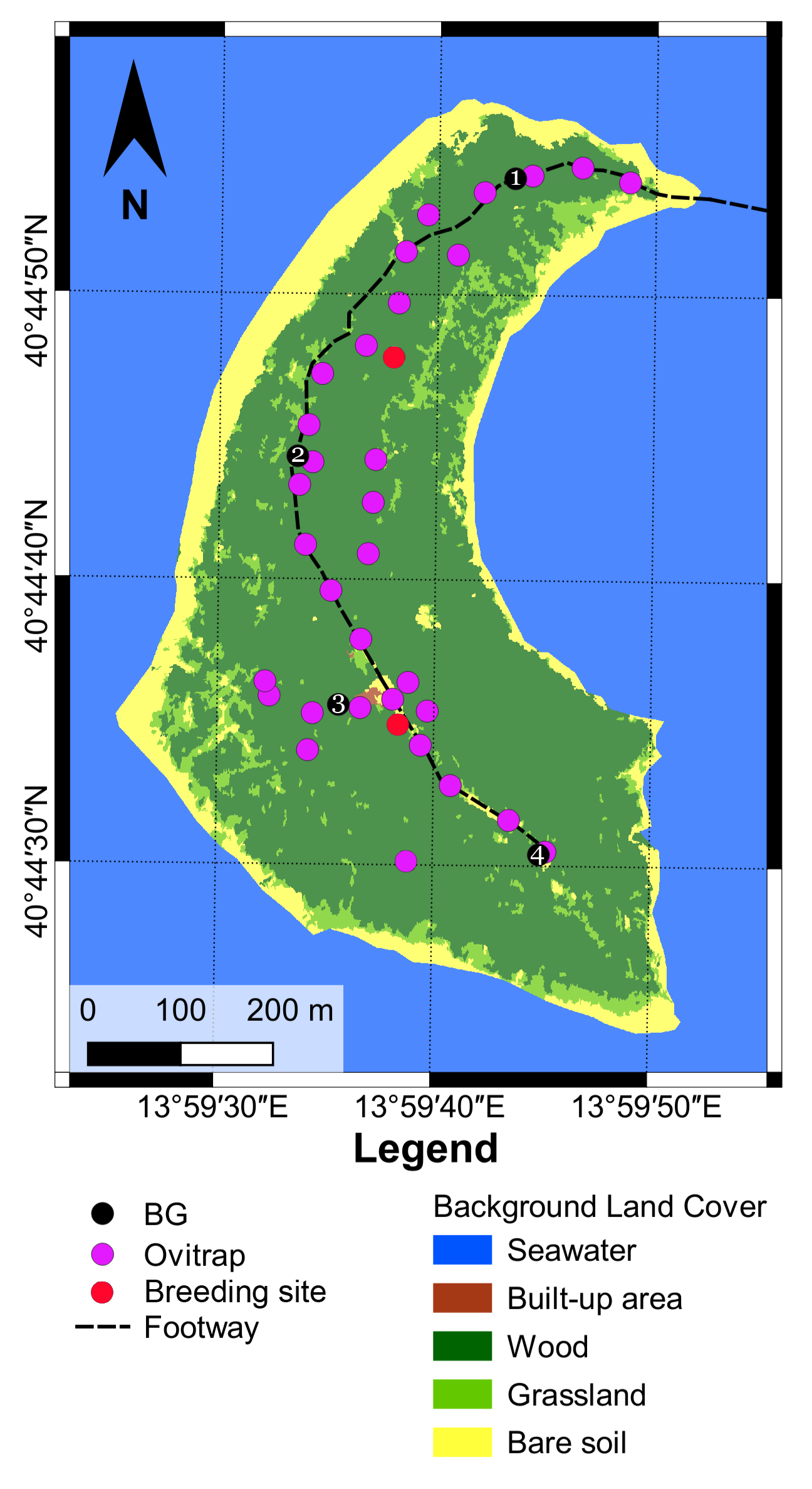

Supplement: S5 Fig — Purple circles represent the position of the ovitraps. Black circles represent the position of BG-sentinel traps. Red circles indicate the position of two identified breeding sites, in both cases represented by ancient cisterns for the collection of rainwater. The base layer of the geographic background map has been sourced from an open maps access (https://glovis.usgs.gov/app). (TIF) [file pntd.0009698.s005.tif]

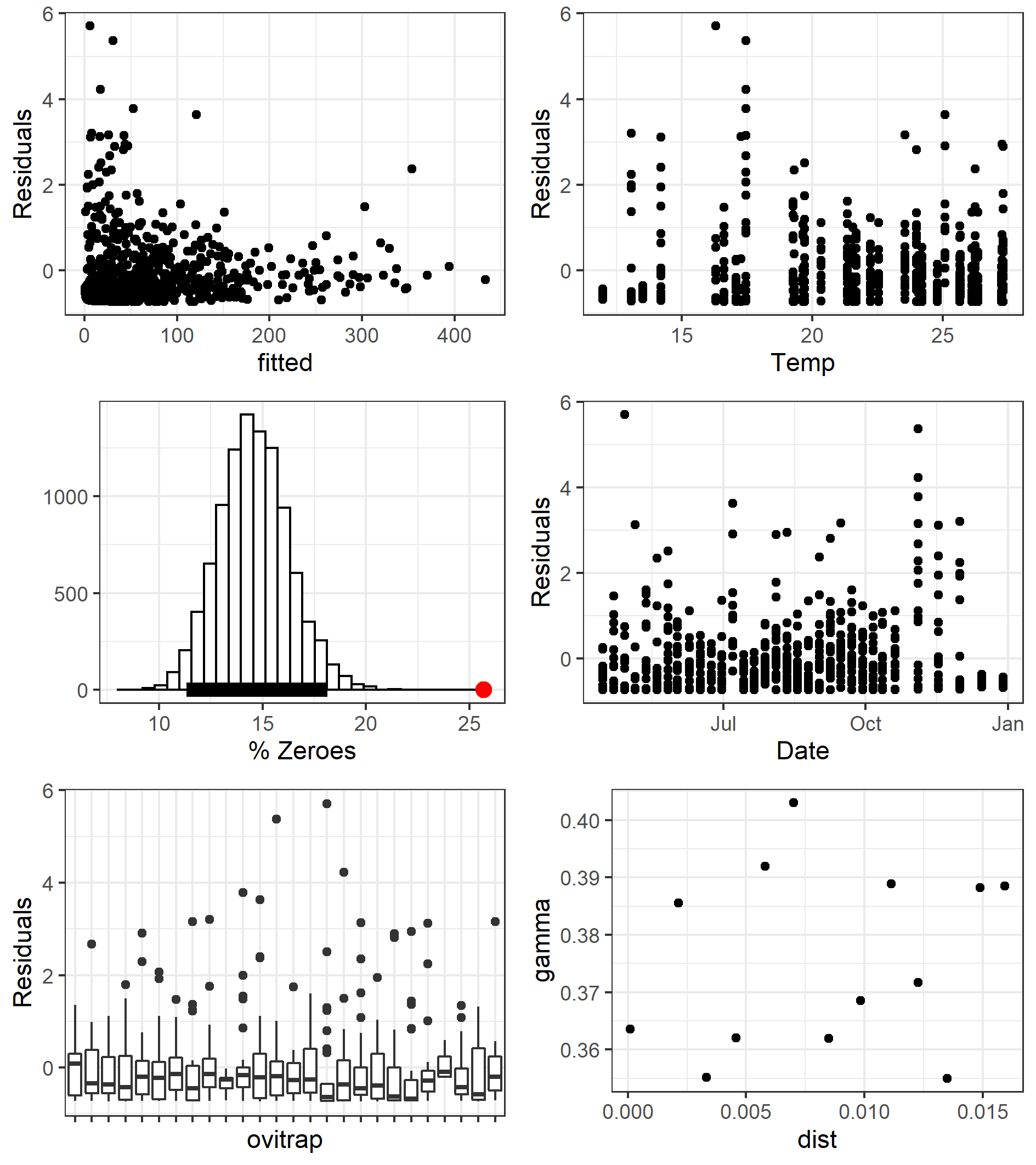

Supplement: S6 Fig — Upper right panel: Pearson’s residuals versus fitted values. Upper left panel: Pearson’s residuals versus temperature. Middle right panel: Histogram of % of zeroes obtained by simulating 10000 databases, the red dot represents the observed % of zeroes. Middle left panel: Pearson’s residuals versus fitted date of collection. Lower right: Pearson’s residuals versus ovitraps. Lower left: Variogram of Pearson’s residuals. Autocorrelation function of each ovitrap time series did not show serious violation of independence. (TIF) [file pntd.0009698.s006.tif]

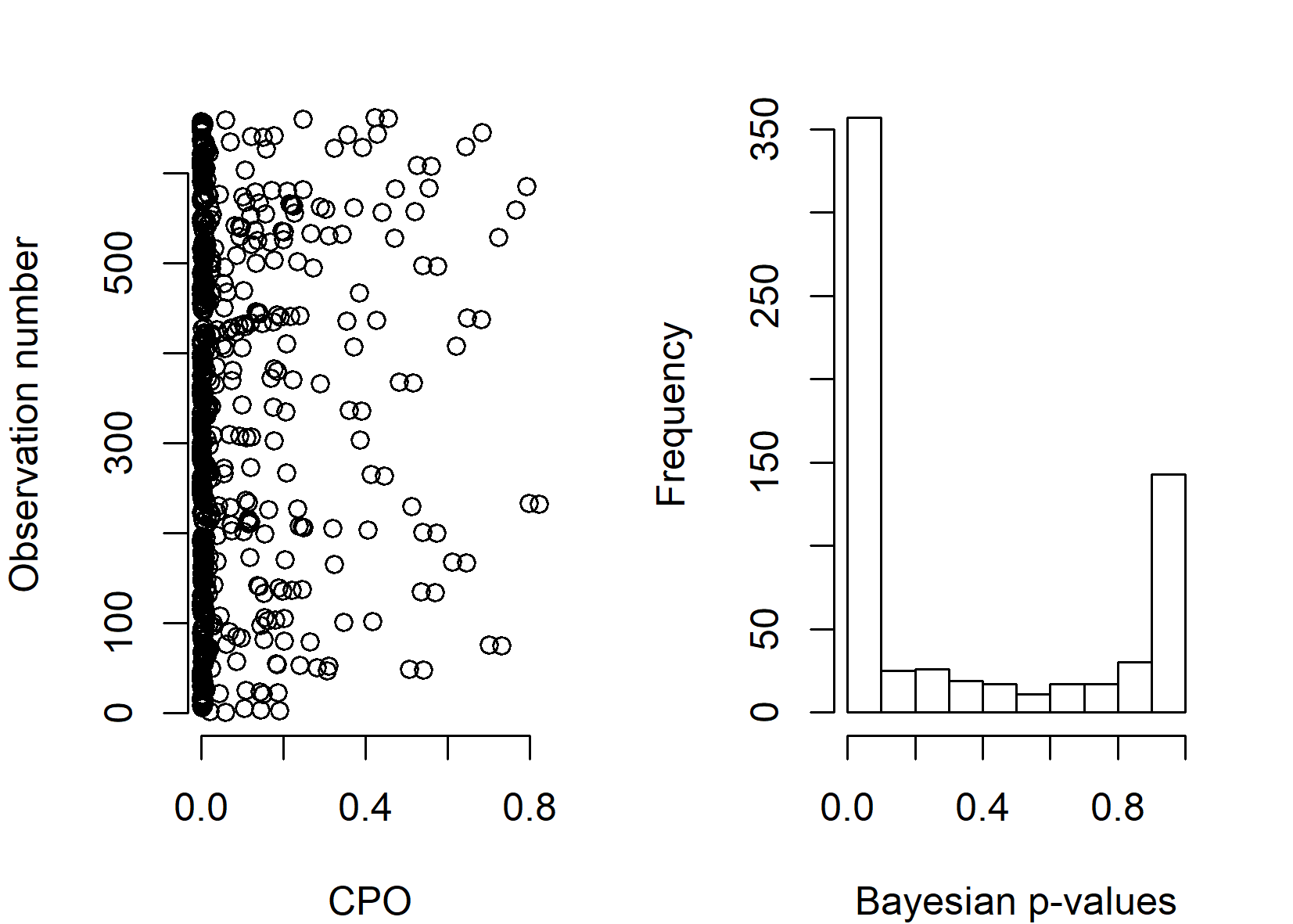

Supplement: S7 Fig — Left panel: On the x axis the conditional predictive ordinate (CPO) which represents the posterior probability of observing that observation when the model is fit using all data except that one. On the y-axis the observation. Right panel: the frequency distribution of the probability of a new value to be lower than the actual observed value (Bayesian p-value). (TIF) [file pntd.0009698.s007.tif]

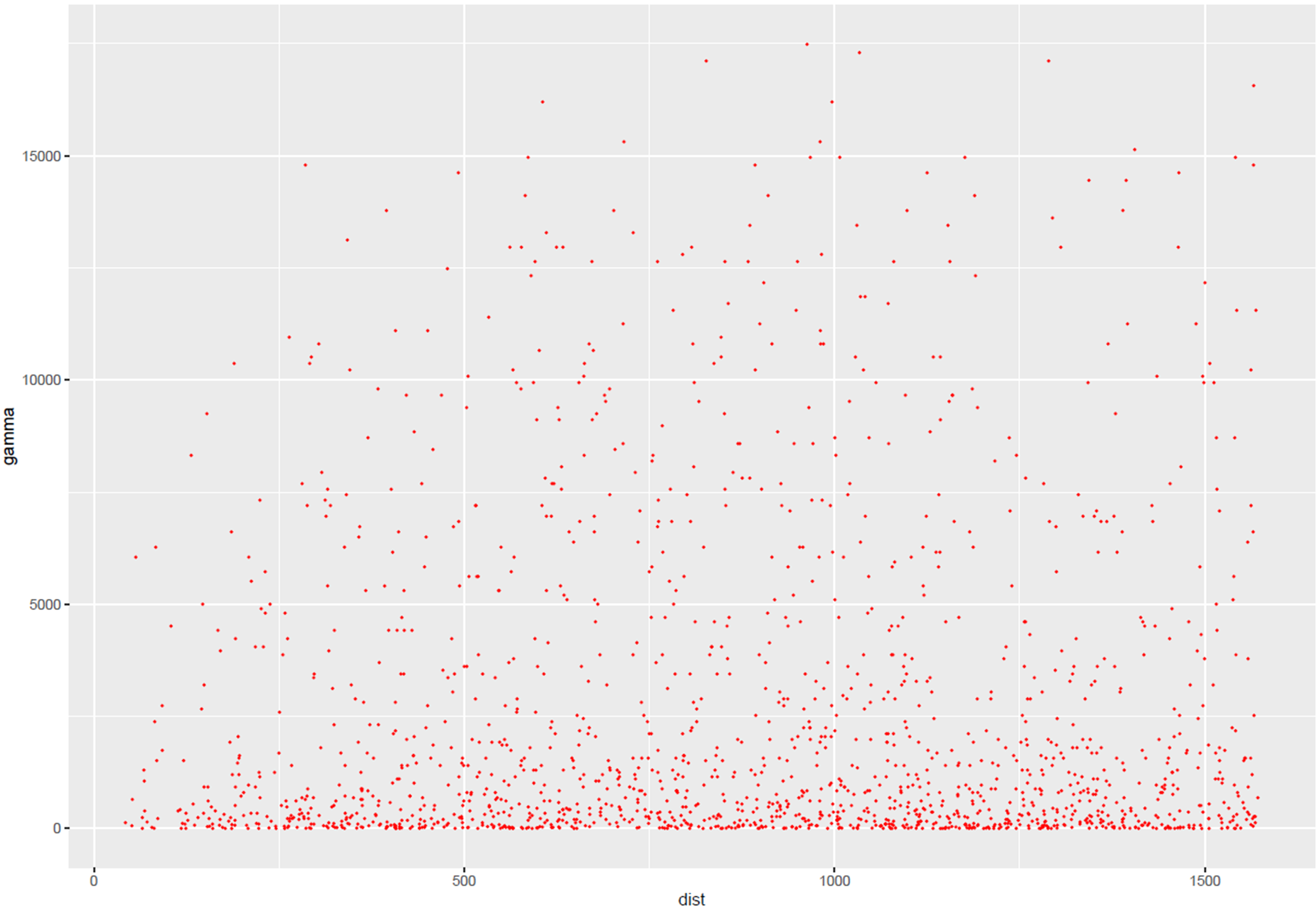

Supplement: S8 Fig — Each point on the plot represents a couple of two locations separated by a distance vector in 2D spatial domain (x axis) and having a semivariance value reported on y axis. (TIF) [file pntd.0009698.s008.tif]

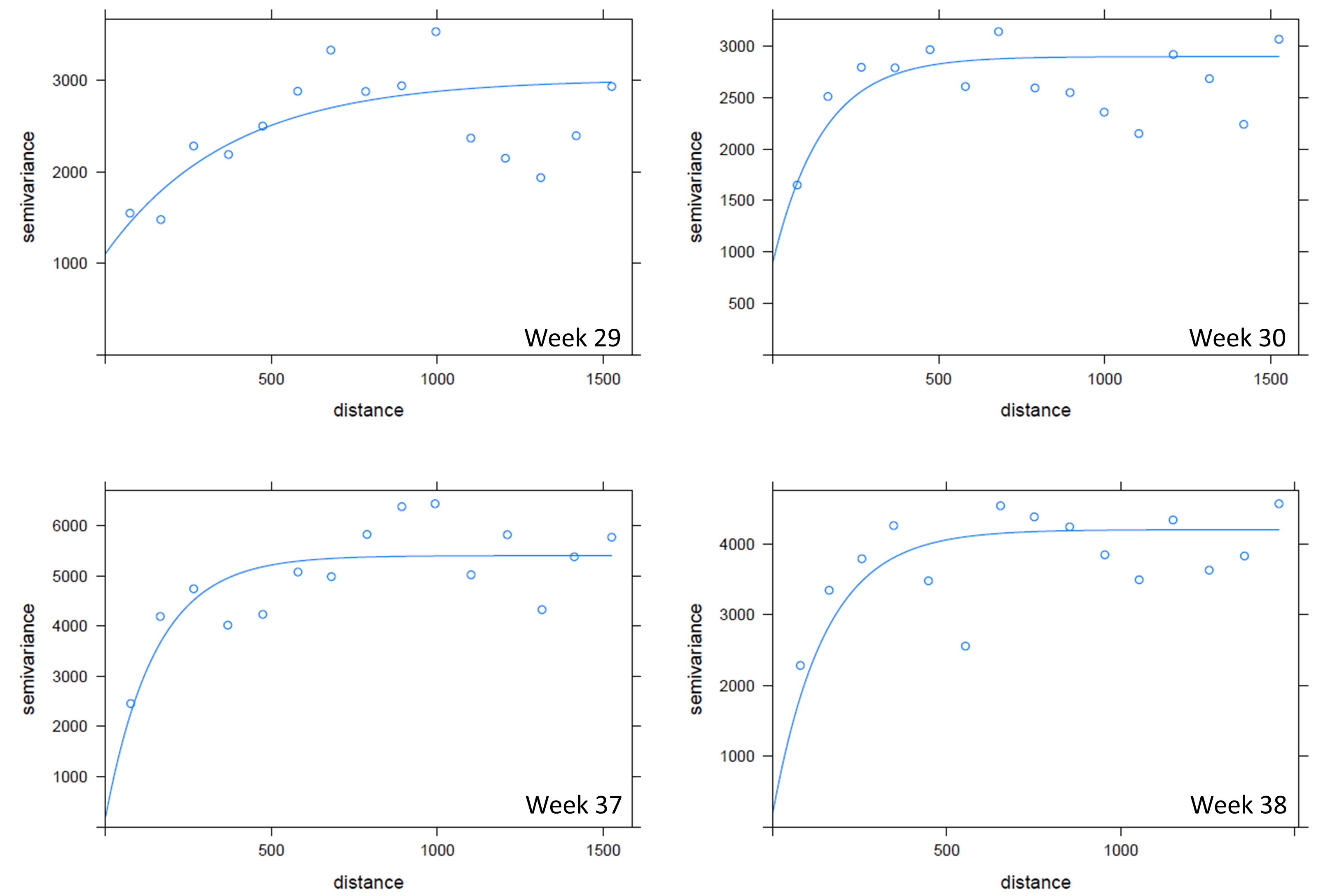

Supplement: S9 Fig — Each point represents the average value of semivariance of couple of locations belonging to the same lag. This is called semivariogram and it is the main tool in geostatistics to discover the existence of spatial structure in the data. It is used to inform the interpolation by kriging. (TIF) [file pntd.0009698.s009.tif]

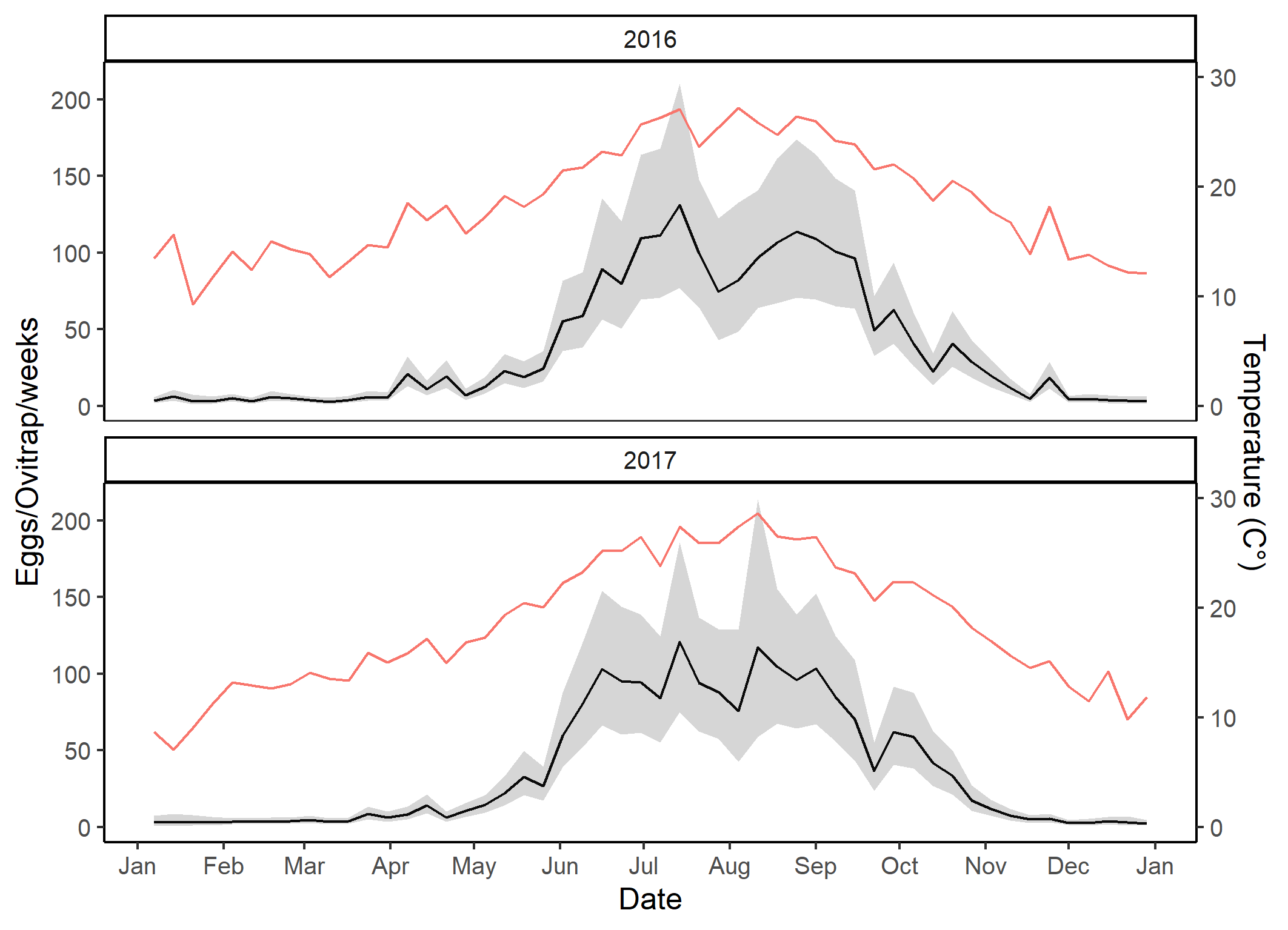

Supplement: S10 Fig — Pattern of Ae. albopictus eggs/ovitrap/week and temperature on Procida Island in 2016 (upper panel) and 2017 (lower panel). On the x-axis the date. The black solid lines represent the GAMM posterior predictive mean value of eggs in ovitrap (left y-axis), the shaded areas represent their 95% credible intervals. The red solid line represents temperatures (right y-axis). In the upper panel the black solid line (the posterior mean) is estimated from observed data (2016). (TIF) [file pntd.0009698.s010.tif]
