# Supplementary material for: Aedes albopictus bionomics data collection by citizen participation on Procida Island, a promising Mediterranean site for the assessment of innovative and community-based integrated pest management methods
Source: PLoS Negl Trop Dis. 2021 Sep 16;15(9):e0009698. doi: 10.1371/journal.pntd.0009698 (PMC8445450; doi:10.1371/journal.pntd.0009698)
Supplement: S1 Text — (DOCX) [file pntd.0009698.s011.docx]

**S1 Text.** **Equations for mean distance travelled computation.**

The equation (14) in the text is obtained from this equation:

$\log\left( \frac{\pi}{1-\pi} \right)=-\log\left( N \right)-log[S_{m}(t)]+log(M)$, (16)

where π is the population fraction of marked mosquitoes, N is the population size, M is the number of mosquitoes released and $S_{m}\left( t \right)$is a survival function. Suppose that survival function is an exponential distribution:

$S_{m}\left( t \right)=e^{-\lambda t}$ , (17)

the equation (14) become:

$\log\left( \frac{\pi}{1-\pi} \right)=-\log\left( N \right)-e^{\beta_{0}}t+log(M)$,

*N* can be estimated from the intercept of the model,

α= $-\log\left( N \right)$; (18)

$\hat{N}=exp(-\hat{\alpha})$. (19)

The approximate 95% confidence interval has been calculated by:

$\exp\left[ -\alpha-zSE\left( \hat{\alpha} \right) \right]<N<\exp\left[ -\alpha+zSE\left( \hat{\alpha} \right) \right],$ (20)

Where *z* is a standard normal distribution.
