# Supplementary material for: Aedes albopictus bionomics data collection by citizen participation on Procida Island, a promising Mediterranean site for the assessment of innovative and community-based integrated pest management methods
Source: PLoS Negl Trop Dis. 2021 Sep 16;15(9):e0009698. doi: 10.1371/journal.pntd.0009698 (PMC8445450; doi:10.1371/journal.pntd.0009698)
Supplement: S2 Text — (DOCX) [file pntd.0009698.s012.docx]

**S2 Text. Equations for expected recapture rate calculation.**

$ER=\frac{N^{\circ} of recaptured in each annulus}{N^{\circ}traps in each annulus}*CF$ , (21)

CF is a correction factor to account for differences in trap densities among annuli

$CF= \frac{\pi(R^{2}-r^{2})}{\pi R^{2}}$*N° of traps in the study areas, (22)

The Mean distance Travelled values was calculated for the first release and for the second release. The Flight Ranges (FRs) were obtained from the linear regression of the cumulative number of expected recapture (ERs) from each annulus on the log_10_ (distance) [1].

**References**

1. Manica M, Filipponi F, D’Alessandro A, Screti A, Neteler M, Rosà R, et al. Spatial and Temporal Hot Spots of Aedes albopictus Abundance inside and outside a South European Metropolitan Area. PLoS Negl Trop Dis. 2016. doi:10.1371/journal.pntd.0004758
