# Supplementary material for: Aedes albopictus bionomics data collection by citizen participation on Procida Island, a promising Mediterranean site for the assessment of innovative and community-based integrated pest management methods
Source: PLoS Negl Trop Dis. 2021 Sep 16;15(9):e0009698. doi: 10.1371/journal.pntd.0009698 (PMC8445450; doi:10.1371/journal.pntd.0009698)
Supplement: Attachment — Submitted filename: Caputo_et_al_2021_response_to_reviewers.docx [file pntd.0009698.s016.docx]

**Responses to reviewers**

-----------------------------------------------------------------------------------

**Methods**

**Reviewer #1:**

Minor revision - additional experiments are not required, but methods section needs improving. An additional experiment assessing the impact of SIT on population abundance, biting pressure, etc would significantly strengthen this paper.

R: We thank the reviewer for his comment. We improved the Methods section, reducing redundancy and increasing clearness. About the opportunity to perform an assessment of the SIT on population abundance and biting pressure, for this study we decided to focus on biological aspect of bionomic of *Aedes albopictus* (seasonal dynamic, dispersal, spatial abundance) and we did not study the impact of control by SIT. We will consider this suggestion for future experiments on the island, and we will try to add these tests to our future program.

**Reviewer #2:**

The objectives of this study were to use a community-based approach to collect baseline data about mosquito bionomics on the island; while no testable hypothesis was stated, it is apparent that the successful collection of biomic data would validate the feasibility of this approach.

R: We thank the reviewer for his comment. Our manuscript is about a descriptive study for future SIT tests for mosquito control by involving community in monitoring mosquito and not testable hypotheses were stated about *Aedes albopictus* bionomics. We expected to obtain for Procida Island *Ae. albopictus* bionomic data similar to the data just produced by other groups in the same Mediterranean temperate area. Despite no novel data have been produced, the info about spatial-temporal dynamics and density of the mosquitos on the island, as well as the dispersal and survival of sterile males in the island environment, are essential premise that we need for future application of SIT. In addition, our results highlight the success of the innovative community-based approach utilized to collect baseline data.

A primary source of data for this manuscript is egg collection from ovitraps, and as such it is crucial that the collection method allowed for accurate egg quantification. In the methods, it is stated that the cylindrical traps (12cm diameter) have an overflow hole at 8cm, yielding a capacity of just under 1L before overflow, and 600mL of water are added before weekly checks-- this starting volume is calculated to come up to ~5.5cm from bottom of the trap, or ~2.5 cm from overflow hole. However, no confirmation is provided that water overflow did not result in the loss of eggs; with 80cm of rainfall per year (methods: study site), several cm of rainfall and subsequent egg loss in a given week would appear to be quite feasible. The methods used to prevent egg loss, accounting for any overflow, and/or confirmation that such loss did not occur should be thoroughly discussed.

R: We thank the reviewer for this comment and we modify the text of the manuscript to clarify this point. The overflow hole in the ovitrap is located at 8cm from the bottom of the trap. As removable substrate for egg collection, we utilized strips of germination paper (30cm x 9cm), lined to the internal wall of the ovitrap. This kind of paper has a typical rippled surface that increase the adhesion of the eggs, preventing their loss. In addition, the height of the strips overcomes the position of the overflow hole, preventing the loss of the eggs by filtering the water in case of overflow.

Several critiques of the MRR methods are raised:

1)	The quantity of fluorescent powder applied to the mosquitoes is not indicated; Culbert et al (PMC7158013) show that Ae albopictus longevity is affected by this marking process at all but the lowest doses tested, which should be accounted for in the analysis.

R: We thank the reviewer for his comment. For this study we did not carry out a particular experiment to test the longevity of marked mosquito. The dose used in this trial (0.3gr/1000 males) to mark the mosquito males at room temperature is the same indicated in the protocol published by FAO/IAEA (2020) in the Guidelines for Mark-Release-Recapture procedures of Aedes mosquitoes(http://www-naweb.iaea.org/nafa/ipc/public/Guidelines-for-MRR-Aedes_v1.0.pdf). The survival of male Ae. albopictus marked with this protocol was not different compared with that of a control group in laboratory conditions. In addition, in a previous works (PMID: 31311945) we carried out a semi-field cages experiment to demonstrate that the survival of marked Ae. albopictus individuals was not affected by fluorescent dyes (Marini et al. 2010). We added this info in the text.

2)	I was unable to locate any information on the fluorescent powder used based on the information provided in the manuscript: while fluorescent dust has been reported to be detectable on mosquitoes for upwards of one month (e.g. PMC71558013), a valuable addition to the experiment would be to have a cage of marked mosquitoes remain outside at the release site (or equivalent environment in regards to wind, rain, humidity, etc) to ensure that dusting was adequate to ensure detection at 13 days. Alternatively, the authors should indicate why they are confident that the dusting was sufficient for detection throughout the study.

R: We thank the reviewer for his comment. The info about the fluorescent powder utilized is present in the Methods section (PROCHIMA s.r.l., Calcinelli di Saltara (PU), Italy). We added in the text the code of the product (PG-661) and the dose of powder utilized for the marking procedure (0.3gr/1000 males). In a previous manuscript (PMID: 20666995), we shown that individuals marked with fluorescent powder were recaptured between days 13 and 17 after release. So, we are confident that the fluorescent powder used was adequate to ensure detection more than the 13 days from the release, as in the current experiment. We added this info in the text.

3)	The use of human landing catches is unexpected given the collection of males, which are not attracted to humans. Please explain the rationale in the methods.

R: Human landing catches have been previously used in other papers to collect *Aedes albopictus* males (ISSNs: 03855643, 00029637; PMIDs: 12597647, 20996630). *Aedes albopictus* males are known to be nearby the human host, attracted by the females. Aedes albopictus is known to mate both nearby the host as well as by producing small swarm. We added this info in the text.

**Reviewer #3:**

In general, my main methodological objection is the apparent lack of geographical and bionomical descriptions: breeding sites are not studied even though they would compete with ovitraps, and land cover is used as a covariate not influencing the mosquito distribution, which could be quite surprising in a less uniform landscape. These topics are somewhat adresses in discussion, but i think they should be introduced from the beginning, very especially about the particular urban configuration of the majority of the land. Taking a view of the island on Google maps is clarifying, I think this kind of graphical information would be necessary from the beginning.

R: We thank the reviewer for his comment. We add a description of the uniform and urbanized configuration of the island in the text. We also add, as graphical information (map of the island with land cover and satellite image) as requested by the reviewer in the supplementary materials (Fig. S1). For what concerns the covariates, as suggest by low Pearson correlation values (Tab S2), all the spatial covariates poorly contribute to the analysis of spatial variance of ovideposition.

L191 - Is really 2.83 inh/Ha a "very high" (human) population density? I feel this is not to be considered really high in comparison to other urbanized areas.

R: We thank the reviewer for this comment. We made a typo error. The correct Procida population density is 27.83 inh/Ha (10298 citizens on 370 Ha). We corrected this data in the text. The Italian average population density is about 1.994 inhabitants/Ha. Italian big cities such as Milan, Rome and Naples have a population density of 20.72, 7.93 and 25.74 inhabitants/Ha, respectively. The average Italian insular population density is 1.35 inhabitants/Ha.

L207 and others - I feel (as the authors actually highlighted in discussion) that the ovitrapping data are a bit poor, both in seasonality (first populations appearing late April whereas the ovitrapping only started mid- April) but also suffering from a single-year sampling.

R: We agree with reviewer. As already pointed in the discussion, our analysis would have benefit from different seasons of monitoring, as well as, from ovitrap activation earlier in the season. However, our temporal data, collected with 26 ovitraps demonstrated that only half of the ovitraps collected eggs in April, and with a very low number of eggs. This suggests that we were able to correctly map the start of the mosquito season in mid-April.

L226 - Is it possible to explain which were the other public activities?

R: We really thank the reviewer for this comment. We added more descriptions about public activities in the text.

L241 - What is the male separation efficiency of this method? Was the number of released females assessed?

R: With this method, males can be separated out with 99.0% accuracy as shown in Balestrino et al. 2014 (PMID: 24647347). However, for this trial particular attention was paid to reduce the female contamination in the released males. Quality control tests carried out on samples of approximately 300 pupae, resulted in 0% of females. We added this info in the text.

L273 - It is quite surprising the use of HLC captures for males since this is a method traditionally used to capture biting females. This should be discussed - if a general, manual male collection is involved, another acronym should be used (and screened areas, such as vegetation, described).

R: The *A. albopictus* males, attracted by females, were collected nearby the human legs of the operator when they are prone to mate with females. We did not perform any male collections within the vegetation. We modify the text to clarify this point.

**Reviewer #4:**

The methods are well designed addressing the objectives of the study, and the statistical analyses is appropriate. Language editing, spelling mistakes need to be corrected.

R: We performed a text editing and revisioning to eliminate mistakes and to improve the written English of our manuscript.

-----------------------------------------------------------------------------------

**Results**

**Reviewer #1**

Major revision - there was a great deal of method repetition in the results section and results were not clearly communicated. The section needs to be restructured with more consideration given to the key results from each piece of the study.

R: We thank the reviewer for this comment. We revised the manuscript according to reviewer suggestion.

**Reviewer #2**

While the analysis presented match the overall goals of this project, several issues with the data presentation arise. Minor modifications are suggested in the “Editorial and Data Presentation Modifications” section, while more significant requests are listed here:

Figure 1:

Since the results in figure 1 are predictions based on a model based on collected data, it would be useful to see the collected data on which the model was based as one panel, and the model predictions (currently the left panel) as a second panel.

R: As suggest by the reviewer, we introduce the observed values into the figure 1 (Black dots = observed values of eggs/ovitrap/week) both for temperature and for cumulative rainfall panels.

Figure 2:

The text refers to Fig 2 only briefly and when describing the relationship between predicted and observed values and should more fully describe the 26 panels and the relevance of the model being accurate across all sample locations. Further, it is not clear whether the Pearson’s correlation indicating a positive correlation are for any of the 26 sample locations, all of them taken collectively, or the data in figure S3.

R: Many thanks for the suggestions. We introduce a description for the relationship between predicted and observed values for some sampling site of the 26 panels. The Pearson’s correlation indicates a positive correlation of overall sampling.

Figure 3:

It is recommended that the authors consider revising this figure to more clearly convey the relationship between temperature and egg deposition, by combining the independent (temperature) and dependent (eggs) variables onto a single graph. Two panels for the two years are suggested since the years are fundamentally different, in that 2016 is based on observed data, and 2017 is a prediction.

R: We thank the reviewer for this comment. The relationship between temperature and egg deposition is already shown in figure 1. Therefore, to shorten the manuscript and avoid confusion we decided to move Figure 3 in the supplementary materials (as Fig. S10) after accepting all improvements suggested by the reviewer. In particular for this figure, according to the reviewer’s request, we decide to split it in two panels one for 2016 (temperature and observed data) and one for 2017 (temperature and predicted data).

Figure 4:

The shades of blue and circle size are unclear: the description of “week” above is difficult to understand, as each panel appears to refer to a single week. The difference between color and size of circle is not indicated; if they are redundant, this redundancy should be eliminated or described. The numbers (e.g., 100, 500) have no units, I believe this refers to eggs/ovitrap, but this should be indicated. Finally, all four panels should use the same scale, since they are quite similar, and the top right panel should have the color/size legend flipped to match the rest. In all, this figure needs substantial work to be comprehendible by the reader.

R: We thank the reviewer for this comment. According to the reviewer we corrected the term “week” with “eggs/ovitrap”. We decide to maintain the redundant double scale (circle size and colour point) to better visualize the differences between overlapping traps. We revised the figure by deleting the four legends and by maintaining the same scale for the four panels, as requested by the reviewer.

**Reviewer #3**

Most results are conforming to what one could expect. Spatial analysis would benefit from an analysis on the distribution of natural breeding sites - similarly, in Vivara reserve breeding sites should be identified (are Tiger mosquitoes present there in high densities coming from mainland?).

R: We thank the reviewer for this comment. In Procida island there is a wide presence of potential breeding sites created by human activities mostly related to cultivation of plants in orchards (we added this info in the Methods section). A spatial analysis of the presence/active breeding sites is not feasible because of their huge number and their possible changes in time and space over time. We expect that the presence of *Aedes albopictus* breeding sites is widespread in island and we do not expect a different competition between ovitraps and natural breeding sites depending on the geographical unit because of the uniform habitat in the main territory of the island. However, this is different in Vivara island, where no human lives, and not human activities are carried out. Vivara represent a natural reserve and in our field exploration we identified just two breeding sites represented by ancient water tanks. We add the position of the two natural breeding sites identified in Vivara in the Fig S6. Vivara island is connected with Procida island through a concrete bridge, 150mt long. The bridge area is exposed to high wind during most of the day. Hence, only sporadic A. albopictus immigration can be envisioned.

Table 4 - when expressing estimates of "natural" Aedes albopictus populations I guess these are only male populations, like the MRR ones.

R: We thank the reviewer for this comment. As indicates in the legend of the Table 4 and in the main text, the population size estimated by the model was the male population. To help readers we change accordingly the legend and the Y-axis of the Figure 7.

**Reviewer #4**

The analysis of the study is sufficient and meet the analysis plan, the results are also clearly presented, spelling and punctuations need to be revised.

R: We agree with the reviewer and we revised the spelling and punctations of the manuscript according with the request.

-----------------------------------------------------------------------------------

**Conclusions**

**Reviewer #1**

Major revision - The discussion section only weakly discusses the paper’s findings in context of other literature. It is clear that there is value to the work that was conducted, but it is not framed and discussed appropriately throughout the discussion section. I suggest bulking that section up and really connecting your research to other’s. It will have the most impact when it is considered amongst the broader database of research on the topic.

R: We thank the reviewer for this helpful comment. We modified the discussion including the most recent literature and improving the discussion of our data (community-based approach, dynamic, population size, habitat suitability) in the frame of the most recent publications in this field.

**Reviewer #2**

The conclusions are supported by the data presented, and the authors make excellent points in the Discussion regarding the applicability of this work to future pest management efforts.

R: We thank the reviewer for his encouraging comment.

**Reviewer #3**

Discussion and conclusions are adequate as they also underline (though for the first time across all the manuscript) some of the methodological weaknesses: egg monitoring for only one season, assumptions on the MRR approach. I would add here a mention on the breeding sites distribution, maybe across L687-L690 where a massive and uniform presence of Ae.albopictus is claimed. Most of this, however, should not be placed on discussion but in Material and Methods because it is a description of the study area which should come forefront.

R: We thank the reviewer for the positive comment. According to the reviewer’ suggestions, we add in Method section a brief description of Aedes albopictus potential breeding sites on the island.

**Reviewer #4**

The conclusion Final conclusions of the paper are too general, some sentences suits the discussion rather than the conclusion. It needs to be more precise and concise. recommendations for similar future studies favoured to be added.

R: We thank the reviewer for this comment. We modified the text according with the reviewer suggestion.

-----------------------------------------------------------------------------------

**Editorial and Data Presentation Modifications**

**Reviewer #1:**

Minor revision - primarily editorial. Most figures are displayed in a clear way that is easy to understand.

R: We thank the reviewer for the positive comment.

**Reviewer #2:**

I don’t have a strong modelling background and thus cannot comment on the appropriateness of the models or presentation; these may be of limited interest to the average reader, and the specifics of the models may be better suited for supplemental materials.

R: We understand that for PNTD readers modelling could be a difficult topic not easily appreciable, but we believe that a brief description of modelling background in Methods section is necessary for a better understanding of the benefits and limitations of the utilized models. However, to help the readers most of not necessary details of the model output have been already inserted in the supplementary material (see Supplementary Fig. S6 to S9).

In the methods (citizen science section), the period of Sept 2015-Sept 2018 is indicated as a four-year span (that period is only three years).

R: We corrected the text accordingly (four years 2015-2019)

The discussion refers to the second survey taking place in 2018, while the results section lists 2019; please align to the correct year.

R: We corrected the text accordingly (2019)

Figure S8 does not contribute appreciably to the manuscript and should be removed.

R: We agree with the reviewer that this supplementary figure (now fig. S3) does not contribute to the paper in terms of data or results. At the same time, we think that it is a nice acknowledgement to our volunteers and that it could help in motivating future volunteers to contribute to the active monitoring of mosquitoes also in other future scientific work. For this reason, we prefer to have this figure in the final version of our manuscript. We changed the order of the figures and FigS8 is now FigS3 in the revised manuscript.

Figure 1:

-Requires units in the x-axis of both panels

R: We thank the reviewer for this comment. We introduced in the figure the units of both axes.

Figure 4:

-Requires units for the y-axis of the upper panel.

R: We thank the reviewer for this comment. We modify the Figure accordingly.

Table 3:

Requires a unit for all numbers: meters?

R: We thank the reviewer for this comment. We introduce it (meters) in the legend of the Table 3.

**Reviewer #3**

L99 - Spanish event was not Chikungunya but Dengue, need to change that

R: We thank the reviewer for this comment. We revised this error according to the reviewer.

L106 - Using mainly chemical pesticides should perhaps be framed as an Italian topic. In several other EU countries the Tiger mosquito is being dealt with by massive microbian larviciding, which additionally should merit some discussion in this paragraph.

R: We thank the reviewer for this comment. We revised it according to the reviewer.

L112 - On the description of SIT or IIT I would prefer to always use the adjective "experimental" complementary control methods. These techniques exist and are being tested since decades on mosquitoes but haven't yet been on practical use.

R: We agree with the adjective "experimental”, and we think that more research is needed. In this light, this work represents a baseline data for future experimental work by SIT or IIT. We revised it according to the reviewer.

**Reviewer #4**

The paper is presenting an implementation research towards Aedes albopictus the principal vector of arboviruses and dengue control. The introduction needs a lot of language editing. After some minor revision I think the paper will be suitable to publish in your precious journal.

R: We thank the reviewer. We revised the main text of our manuscript to improve its readability.

-----------------------------------------------------------------------------------

**Summary and General Comments**

**Reviewer #1**

To the authors,

Thank you for contributing your work to PLOS NTD. It is clear that an immense amount of work went into this project and it was truly a collaborative effort. I hope that my comments below and in my marked pdf aid you in improving the manuscript.

There is certainly scientific rigor in the research you conducted, but it’s communication in this manuscript needs significant improvements. If this is done, I have no doubt that it will improve the clarity and readability of the paper and allow it to reach a broader scientific community.

In general, I believe the tone of the paper is at times too casual. The authors did a great job of providing detail and resources, but phrases like “Thanks to their help” and other similar phrases should not be used. There are more formal ways of making these points that are appropriate for scientific papers.

I also think the authors would benefit from using more concise language wherever possible. There are many extra words used throughout the paper that can be eliminated. The word very was used a lot, for example. I would suggest having a fresh set of eyes read through before submitting the revision. It just needs tightening up.

At some points, I feel the level of detailed provided exceeds what is traditionally included in a manuscript. Much of that information can be removed and simplified.

The results section feels very unclear. I don’t feel like the major finding from analysis or trends in the data were discussed in an understandable way. It’s difficult to read as currently written and there are pieces that are more fitted for other sections of the paper. I have tried to point some examples out, but it can be restructured to benefit the readability and clarity of the paper.

The discussion section only weakly discusses the paper’s findings in context of other literature. It is clear that there is value to the work that was conducted, but it is not framed and discussed appropriately throughout the discussion section. I suggest bulking that section up and really connecting your research to other’s. It will have the most impact when it is considered amongst the broader database of research on the topic.

Overall, I would like to repeat my suggestion to have a fresh set of eyes read through the paper. It needs some significant restructuring and can be formalized and trimmed down significantly. Despite these needs for the paper, I still believe the work to be valuable and worthy of publication, but only with these significant improvements.

My specific comments are included in the pdf but are by no means exhaustive or all inclusive. Consider my comments in context to the entire paper, not just the pieces I called attention to. Best of luck with revisions.

R: We are deeply grateful to the reviewer for his encouragement and for the effort done in the editing of our manuscript. We accepted all the correction/suggestion in the edited pdf file, and we modified the text accordingly.

**Reviewer #2**

This manuscript provides a useful description of population characteristics of the Ae. albopictus mosquito on Procida, Italy, and the utility of this small Italian island for pest management studies. Further, the authors went to great lengths to involve the local community in the execution of this research and have presented a convincing model for a community-based approach to mosquito management. The authors show that the Ae. albopictus egg dynamics are temperature-dependent, weighted towards the end of the summer, are uniformly distributed across the island, and that male dispersal is generally <60m and daily survival is around 0.8-0.95.

While the findings are novel for this particular location, and this island provides a useful tool for future pest management studies, none of the findings add substantial knowledge to the field of vector biology, as all results have been previously reported for Ae. albopictus in other locations, including mainland Italy (e.g. PMC7954105 for temperature-dependence of egg laying in Italy and weight towards the end-of-summer, PMC5669009 for distribution of mosquitoes on Italian islands, PMC6371565 for similar daily survival and dispersal distances).

Some concerns regarding the methods data presentation would warrant revision prior to suitability of this study for publication.

Finally, the language and grammar of the manuscript requires extensive revision to be publication-worthy, and I advise that the authors work with a writing coach to ensure that the manuscript complies with the PLOS NITD requirement to be presented in standard English. Frequent errors in word choice, sentence structure, and mistakes in singular/pluralization as well as un-defined abbreviations require attention. Substantial clarification requests for most figures, such as axis units and description of gradients, emphasize the need for additional editorial work.

R: We thank the reviewer. We modified the text accordingly to his suggestions.

**Reviewer #3**

This is a study dealing with the description of an Asian Tiger mosquito population on an island location which is really adequate as a test field for SIT and any other control technique. The work is well designed in view of the aimed goals, though the experimental base data is in my opinion somewhat too light to support conclusions drawn from quite advanced modelling statistics. In general, no big surprises over what was to be expected, resulting anyway in interesting population data and conclusions.

Citizenship involvment has been sought and obtained from a number of individuals to help in trapping and is highllighted by the authors as a crucial asset in future operations but is not thoroughly described - some notes on what was carried out in this area would be welcome, especially regarding other awareness actions, furthermore considering the survey results were quite poor in terms of user engagement.

We believe this work can be published with some extra clarification.

R: We really thank the reviewer for his comments. We modified the text, also adding more info about the activities performed with citizens. This represents a key point of our approach and in the previous version of our manuscript we completely missed the right description of it.

**Reviewer #4**

The paper revealed a nice piece of implemented research. The author summary and introduction included many misspellings, imperfect and unclear sentences, from my perspective extensive language editing need to be conducted prior publication.

R: We thank the reviewer. We modified and corrected the text accordingly.
