# Supplementary material for: Aedes albopictus bionomics data collection by citizen participation on Procida Island, a promising Mediterranean site for the assessment of innovative and community-based integrated pest management methods
Source: PLoS Negl Trop Dis. 2021 Sep 16;15(9):e0009698. doi: 10.1371/journal.pntd.0009698 (PMC8445450; doi:10.1371/journal.pntd.0009698)
Supplement: Attachment — Submitted filename: Caputo_et_al_2021_response_to_reviewers_v2.docx [file pntd.0009698.s017.docx]

**Responses to reviewers**

-----------------------------------------------------------------------------------

**Methods**

**Reviewer #1**:

Yes, the authors have made significant improvements to the text. The methods are more appropriately described now.

R: We thank for this comment.

**Reviewer #2**:

The critiques I raised about the methods were adequately addressed by the authors

R: We thank for this comment.

**Reviewer #4**:

The methods were improved and corrected following reviewers comments. The methods are well designed addressing the objectives of the study, and the statistical analyses is appropriate. Language editing, spelling mistakes need to be corrected.

R: We thank for this comment. We re-edited the text to further correct spelling mistakes.

-----------------------------------------------------------------------------------

**Results**

**Reviewer #1:**

Yes, this section has also been improved and the information is completely presented.

R: We thank for this comment.

**Reviewer #2:**

The requested edits and clarifications to the results section have been appropriately incorporated into the revised manuscript.

R: We thank for this comment.

**Reviewer #4:**

The results are clearly presented. Table 1 the title is not clear, it needs to be more clear (ex: is it a community survey?), it is also better if you put the title up in the table and the legend in bottom. The tables are not in the same design and layout, please correct.

R: We thank for this comment. We corrected the title of table 1 and we modified the layout of table 1, using the design of the other tables of the MS.

-----------------------------------------------------------------------------------

**Conclusions**

**Reviewer #1:**

Conclusions are concise and represent the major findings of the paper.

R: We thank for this comment.

**Reviewer #2:**

No further recommendations

R: We thank for this comment.

**Reviewer #4:**

The conclusion are supported by the presented results, however the limitations and recommendations to the future research in the filed were not sufficiently addressed.

R: We thank for this comment. As previously requested by reviewers, we modified in our previous revision the conclusion section to have it in a more concise form. For this reason, we preferred to address the limitations of our study in the discussion section. According with the reviewer’s comment, here we modified the conclusion by adding recommendations to the future research in the field.

-----------------------------------------------------------------------------------

**Editorial and Data Presentation Modifications?**

**Reviewer #1:**

Could still use some minor grammar editing but is otherwise much improved from the original submission.

R: We thank for this comment. We re-edited the text to further correct spelling mistakes.

**Reviewer #2:**

No further recommendations

R: We thank for this comment.

**Reviewer #4:**

I think the authors need to modify the manuscript title to be more strong, clear and represent the study accurately.

R: We thank for this comment. We think that the title contains the two main messages of our paper: the production of bionomics data by collaborating with citizen and the focus on Procida island as a promising Mediterranean site for the assessment of innovative and community-based integrated pest management methods. According with the reviewer comment, we slightly modify the title to make it clearer.

-----------------------------------------------------------------------------------

**Summary and General Comments**

**Reviewer #1:**

The authors made significant improvements to the paper that improve its clarity and readability. Identifying all changes without the track changes document was difficult, but the paper is more logically presented now.

R: We thank for this comment.

**Reviewer #2:**

The authors have substantially revised the manuscript to be readable and have addressed the concerns that I listed in my original review. The article is now recommended for publication. Throughout the manuscript, including the title, "in Procida island" is used when "on Procida island" may be more appropriate. The authors should not exclusively use the pronouns 'he' and 'his' when responding to reviewer comments, but rather use statements like "we thank the reviewer for their comment".

R: We thank for this comment. We made the correction about the right use of “on Procida island” throughout the manuscript. We are very sorry for the exclusive use of “his” pronoun in the previous reply to reviewers.

**Reviewer #4:**

After the authors consider all the comments the manuscript will be ready to publish in the journal. I think the authors need to modify the manuscript title to be more strong, clear and represent the study accurately.

R: We thank for this comment. We think that the title contains the two main messages of our paper: the production of bionomics data by collaborating with citizen and the focus on Procida island as a promising Mediterranean site for the assessment of innovative and community-based integrated pest management methods. According with the reviewer comment, we slightly modify the title to make it clearer.
